# How Passion for Playing World of Warcraft Predicts In-Game Social Capital, Loneliness, and Wellbeing

**DOI:** 10.3389/fpsyg.2020.02165

**Published:** 2020-09-15

**Authors:** Regan L. Mandryk, Julian Frommel, Ashley Armstrong, Daniel Johnson

**Affiliations:** ^1^Department of Computer Science, University of Saskatchewan, Saskatoon, SK, Canada; ^2^School of Computer Science, Queensland University of Technology, Brisbane, QLD, Australia

**Keywords:** passion, social capital, loneliness, wellbeing, social games, benefits, problematic gaming

## Abstract

Playing digital games can nurture wellbeing by helping players recover from daily stressors, cope with life's challenges, practice emotion regulation, and engage in meaningful social interaction; however, this same leisure activity can also result in problematic gaming (i.e., harmful play at the expense of healthy behaviors), and social isolation that damages wellbeing. Research consistently demonstrates that the value or harm of gaming on wellbeing cannot be determined solely from whether and how much people play, but rather depends on contingent factors related to the player, the game, and the gaming context. In this paper, we aim to model contingent factors that differentiate between beneficial and harmful outcomes within players of the same massively multiplayer online role playing game (MMORPG). We model how passion for gaming—defined as a strong desire to engage in a beloved activity that is enjoyed and valued, in which time and energy is invested, and that ultimately integrates into a person's identity—affects loneliness and wellbeing. We employ the dualistic model that divides passion into harmonious passion (HP)—characterized by a balanced and authentic relationship with the beloved activity, and obsessive passion (OP)—characterized by preoccupation and inflexible persistence toward the loved activity. We sampled 300 frequent World of Warcraft (WoW) players, recruited from online forums, and used structural equation modeling (SEM) to investigate the effects of their passion for playing WoW on in-game social capital, loneliness, and wellbeing. We demonstrate that HP for playing WoW facilitates in-game social capital (both bridging and bonding), combats loneliness, and increases wellbeing, whereas OP also builds social capital, but these social ties do not combat loneliness, and OP is directly associated with increased loneliness. Further, the positive effect of HP on wellbeing is mediated through an increase in bonding social capital and a resulting decrease in loneliness. Our findings highlight that passion orientation is important for characterizing the relationship between gaming and wellbeing. We contribute to the conversation on combating problematic gaming, while also promoting digital gaming as an appealing leisure activity that provides enjoyment, recovery, and meaningful social interaction for the millions of gamers who benefit from its captivation.

## 1. Introduction

Digital gaming is quickly becoming a leading leisure activity among a broad range of demographics; in 2019, approximately two thirds of the global online population played digital games (Newzoo, 2019). Gamers play on dedicated gaming consoles and on personal computers in their homes, but also on tablets and smartphones out in the world, and in dedicated gaming spaces (Entertainment Software Association, 2019). Research has demonstrated that people play digital games as a leisure activity to experience enjoyment (Boyle et al., 2012), escapism (Grove et al., 2016), immersion (Jennett et al., 2008), and challenge (Denisova et al., 2020), but gaming also provides more than just a pleasurable pastime. Playing digital games has also been shown to nurture wellbeing by helping players recover from daily stressors (Reinecke, 2009; Collins et al., 2019), repair noxious moods (Bowman and Tamborini, 2012), cope with life's challenges (Iacovides and Mekler, 2019), and practice emotional regulation (Granic et al., 2014). In addition to these benefits to the individual player, gaming has also been shown to be socially motivated for both adults (Frostling-Henningsson, 2009) and youth (Ferguson and Olson, 2012). The majority of adult gamers in 2019 played in multiplayer mode with others for an average of 4.8 h per week online and 3.5 h per week in person because they feel that video games help them connect with friends and family (Entertainment Software Association, 2019). For example, Williams et al. (2006) show that World of Warcraft (WoW) players use the game as a platform to maintain existing relationships, form new ones, and even to find romantic partners.

This rise of social gaming coincides with a timeframe in which an increasing lack of social connectedness has been identified as a threat to our wellbeing (OECD, 2020). The need to form lasting and caring relationships and the feeling of belonging are fundamental human needs (Baumeister and Leary, 1995; Deci and Ryan, 2000), but the social connectedness of people has declined over the past decade with the share of people who have relatives or friends they feel that they can count on to help in a time of need having fallen across many developed nations (OECD, 2020). Good social embeddedness is key for overall health and wellbeing; research has shown that when people feel socially excluded, they are subject to impaired executive functioning (Baumeister et al., 2002) and an increased tendency toward hostile cognitions, including the interpretation of ambiguous situations in a threatening way (DeWall et al., 2009). Loneliness is a major contributor to reduced wellbeing, with estimates suggesting that people who feel isolated have a 30% increased risk of mortality (Holt-Lunstad et al., 2015). To help address the more than 9 million residents who often or always feel lonely in the UK, a Minister of Loneliness was appointed in 2018 (Holt-Lunstad et al., 2015). The prevalence of loneliness is highest among 18–30 year olds (Holt-Lunstad et al., 2015)—the same age range of adults with the highest proportion of gamers (Entertainment Software Association, 2019).

Given the increasing prevalence of multiplayer digital games as a leisure activity, in a context of decreasing social embeddedness, researchers have started to consider whether the social relationships that are established and enacted through digital games help or harm social aspects of wellbeing, including loneliness and feelings of isolation. Recent studies have demonstrated that digital games—played both in-person and online—can facilitate social interactions that are vital for our social well-being, for example, by connecting us to others (Dabbish, 2008; Hernandez et al., 2014), helping us maintain existing relationships (Wohn et al., 2011), facilitating trust development with strangers (Depping et al., 2016; Depping and Mandryk, 2017), and even combating loneliness (Depping et al., 2018); however, the same mechanics, games, and gaming contexts that foster social closeness in games can instead lead to toxic game environments (Chen et al., 2009; Kwak et al., 2015) or displace offline relationships (Zhong, 2011), resulting in feelings of social exclusion (Shores et al., 2014).

The potential value or damage that results from social game play can have a great impact on players, but existing contrasting evidence makes it challenging to reconcile the potential benefits and harms of social play on wellbeing. Further, research consistently demonstrates that effects of social play on wellbeing cannot be determined simply from whether and how much people play, but instead depends on a variety of contingent factors, such as how satisfied the individual player is with their life (Przybylski et al., 2009), at what time of day they tend to play (Lemola et al., 2011), and what types of games they choose (Depping et al., 2018). In the context of social play, the role of contingent factors in determining the benefits or harms is still not well understood. Little research has teased out these relationships in the context of social gaming. The problem is that *without an understanding of how these contingent factors influence player wellbeing, we could be encouraging social play with the intention of helping players, but actually harm them; conversely, we might discourage social play to protect players that would actually benefit greatly from participating in game-based social interactions*. There are a variety of contingent factors that might affect the social value of digital game play, including factors related to the player characteristics, the game features, and the experience of play itself (Johnson et al., 2013). When we discuss the impact of social play on wellbeing, we must think beyond simply whether or not people play with others, but rather consider the quality of that social interaction. When considering the quality or value of social interactions, we can turn to the well-established construct of social capital, which helps us situate game-based social interactions in a broader theoretical grounding of the value of social ties.

### 1.1. Social Capital in Games

The social capital framework formalizes the value of social ties, framing social networks as resources that when fostered, will return value to an individual in the form of social support and personal information sharing that benefits wellbeing (Putnam, 2000). The social capital framework differentiates two kinds of relationships: bridging ties and bonding ties (Putnam, 2000). Bridging ties broaden the social horizon of the holder as they expose us to different world views, opinions, and resources (Putnam, 2000; Williams, 2006b); bridging ties are characterized as tentative relationships that may lack depth but make up for it in breadth. In contrast, bonding ties refer to strong relationships in which people feel emotional and social support. Bonding ties are characterized by relationships with less diversity but stronger personal connections, and which provide strong, reciprocated, and substantive emotional support (Putnam, 2000; Williams, 2006b). Social capital is generally associated positively with outcomes related to psychological wellbeing (Putnam, 2000; Williams, 2006b).

Researchers have considered the quality of in-game relationships through the framework of social capital within World of Warcraft (Steinkuehler and Williams, 2006; Williams et al., 2006; Cole and Griffiths, 2007), Second Life (Huvila et al., 2010), and Counter-Strike (Jansz and Martens, 2005; Jansz and Tanis, 2007). Early research (conducted between 2005 and 2010) showed that relationships enacted within these games are capable of generating social capital, but generally agreed that social gaming was more likely to lead to bridging ties than bonding ties (Steinkuehler and Williams, 2006; Williams et al., 2006; Huvila et al., 2010). Subsequent research (conducted between 2011 and 2015) started to investigate how in-game social capital is formed by considering the motivations of players (Shen and Williams, 2011; Domahidi et al., 2014), their play duration and frequency (Shen and Williams, 2011; Domahidi et al., 2014; Kowert et al., 2014a), their physical and social proximity (Trepte et al., 2012), and the intensity of their in-game communication (Shen and Williams, 2011). Recent work (conducted after 2016) further investigated the properties of the game community itself, demonstrating that games requiring interdependence between players and communities with lower toxicity build both bridging and bonding ties (Depping et al., 2018). Finally, Perry et al. (2018) investigated the locus of relationship formation (i.e., whether the gaming relationships were online friends, strangers, or physical world friends) within Destiny players and showed that playing with online and physical friends built bonding ties, whereas playing with online friends and strangers built bridging ties. This research considered not only the type of relationship between players, but also considered how social capital is affected by the type of *engagement* that players had with Destiny, using the Dualistic Model of Passion.

### 1.2. Dualistic Model of Passion

Passion is defined as a strong desire to engage in a beloved activity that is enjoyed and valued, in which time and energy is invested, and that ultimately integrates into a person's identity (Vallerand et al., 2003; Lalande et al., 2017). The passion that people have for activities in their life (e.g., leisure, work, study) affects their engagement with those activities. In a series of studies and within the context of a variety of activities (e.g., work, study, music, sports), Vallerand et al. (2003) explored how passion for an activity develops and manifests, resulting in the Dualistic Model of Passion (DMP). In their work, they discuss how when we engage in an activity, and come to value it, we internalize it and adopt it as part of our self-identity (Vallerand et al., 2003). This process of internalization differentiates simple enjoyment of an activity (or intrinsic motivation to engage with it, Deci and Ryan, 2000) from activities that have become an enduring part of our identity; e.g., gaming as a pleasurable pastime vs. a passion for gaming that has resulted in identification as a “gamer.” The DMP further suggests that a developing passion can manifest in ways that are more harmonious or more obsessive (Vallerand et al., 2003). When people are harmoniously passionate about an activity, they describe it positively, and engage in it freely, authentically, and in balance with other activities or goals in their lives. Obsessive passion also describes activities that we have a strong desire to engage in, but instead desire is characterized as a preoccupation—an uncontrollable urge that is in conflict with other activities and goals and which leads to the neglect of those other pursuits (Vallerand et al., 2003).

Outside of videogames, there is consistent evidence that harmonious passion is associated with increased positive outcomes (e.g., self-development, social interaction, satisfaction with life, vitality) and some evidence that it is also associated with decreased negative outcomes (e.g., negative affect). Conversely, obsessive passion has been found to be consistently associated with increased negative outcomes (e.g., overuse of media, academic burnout, negative affect) and there is also some evidence of decreased positive outcomes (e.g., vitality). The positive impacts of harmonious passion and negative impacts of obsessive passion have been found across contexts including series watching, facebook use, music, sport and work (Vallerand et al., 2006; Lalande et al., 2017; Tóth-Király et al., 2019). The pattern has been found to be equivalent with respect to videogames, with harmonious passion associated with positive outcomes including skill development, motivation to relax and recreate, post-play energy, life-satisfaction and mental health (Przybylski et al., 2009; Tóth-Király et al., 2019), and obsessive passion related to problematic use of videogames, motivation to procrastinate, and post-play tension (Przybylski et al., 2009; Tóth-Király et al., 2019). However, as with other domains, a harmonious passion for videogames has not always been found to be associated with reduced negative outcomes and obsessive passion has not always been shown to be related to reduced positive outcomes. Specifically, Tóth-Király et al. (2019) did not find an association between harmonious passion and decreased overuse of videogames nor did they find any reductions in self-development and social interaction related to obsessive passion.

A recent development in the field is the consideration of passion as a quadripartite construct (Schellenberg et al., 2019). While previously, researchers treated obsessive and harmonious passion independently, the quadripartite model allows for simultaneous consideration of these constructs with people being considered as having pure harmonious passion (high on HP low on OP), pure obsessive passion (high on OP low on HP), mixed passion (moderate to high levels of both HP and OP), and no passion (low levels of both HP and OP). Among undergraduate students considering their favorite activity, pure HP was found to predict the highest levels of global health and psychological wellbeing, while pure OP predicted the lowest levels of both (Schellenberg et al., 2019). Mixed passion and a lack of passion both predicted better outcomes than pure OP. The same pattern was found with respect to passion for study and academic burnout (Schellenberg et al., 2019). With specific regard to passion for videogames, the pattern was largely consistent with pure HP predicting the best and pure OP predicting the worst outcomes in terms of physical health symptoms (e.g., carpal tunnel, dry eyes) (Schellenberg et al., 2019).

### 1.3. Gaming and Wellbeing

Although they entertain us, digital games also offer an opportunity for improving wellbeing, for example by improving emotion regulation (Granic et al., 2014), offering recovery from stress or boredom (Reinecke, 2009; Bowman and Tamborini, 2012), building self-esteem (Bessière et al., 2007), and promoting mindfulness (Collins et al., 2019). Games also allow us to explore difficult emotions (Olson et al., 2008), and help us cope with changing life situations (Iacovides and Mekler, 2019); however, when used as a coping strategy, gaming may also thwart productivity, which creates an obstacle to wellbeing (Iacovides and Mekler, 2019). Wellbeing is also harmed when gaming is associated with addictive behavior (Van Rooij et al., 2011; Griffiths et al., 2012), a lack of control over play behavior (Mandryk and Birk, 2017), or a compulsion to play (Przybylski et al., 2009). However, the lines between engagement and addiction are often blurred, spawning recent efforts to disentangle high passion for gaming that results in beneficial engagement or damaging addictive behavior (Charlton and Danforth, 2007, 2010; Deleuze et al., 2018). The blurred relationships between gaming and its outcomes, which are both beneficial and harmful, has prompted significant research and discussion around the idea of problematic gaming.

The World Health Organization introduced “gaming disorder” as a diagnostic classification into the ICD-11 (World Health Organization, 2018), and while “internet gaming disorder” is not included in the DSM-5 (American Psychiatric Association, 2018), it has been identified as a condition of interest that warrants further clinical research. A gaming disorder is defined as “a pattern of gaming (.) characterized by impaired control over gaming, increasing priority given to gaming over other activities to the extent that gaming takes precedence over other interests and daily activities, and continuation or escalation of gaming despite the occurrence of negative consequences” (World Health Organization, 2018). This characterization of gaming as a disorder has sparked controversy as game scholars (e.g., Griffiths et al., 2016; Aarseth et al., 2017) have raised concerns surrounding its definition, the lack of supporting clinical data, and questions on whether problematic gaming should be viewed as a disorder in itself or as a coping mechanism for a different underlying problem (Kardefelt-Winther, 2014a). Still, problematic gaming has been associated with a range of harms to our physical (e.g., sleep deprivation, day-night reversal, malnutrition/dehydration, seizures), psychological (e.g., depression, anxiety, suicide), and vocational (e.g., impaired work and academic performance) wellbeing (McLean and Griffiths, 2013; Mitchell et al., 2015; González-Bueso et al., 2018; Bányai et al., 2019; O'Farrell et al., 2020). Wellbeing can also be harmed through exposure to toxicity in online gaming, especially when players engage in anonymous and impersonal interactions. Toxic behavior in multiplayer games often takes the form of harassment through verbal abuse (Foo and Koivisto, 2004); however, toxicity is also expressed through any behavior that harms team cohesion, such as negative attitudes toward teammates, refusing to help your team, purposefully losing, or not participating (Chen et al., 2009; Kwak et al., 2015). Toxic behavior not only affects a player's game experience (Shores et al., 2014), but has been shown to thwart the development of in-game social capital (Depping et al., 2018), and also harm wellbeing by leading to depression, anxiety, and even suicide (Kwak et al., 2015). A 2015 study found that 52% of Massively Multiplayer Online Role Playing Game (MMORPG) players had been victims of cyberbullying (Ballard and Welch, 2015). Beyond the exposure to toxicity experienced in online gaming, researchers have also questioned the potential social harms caused by investing in online in-game relationships, at the expense of offline relationships in the physical world.

Early research into the social impact of gaming suggested that online social capital developed through games does not transfer into the physical world (Huvila et al., 2010; Zhong, 2011; Kwak et al., 2015); however, only a few studies have directly investigated the relationship between in-game interactions and offline social embeddedness. Williams conducted a series of studies within the WoW context (Steinkuehler and Williams, 2006; Williams et al., 2006; Shen and Williams, 2011), and reported that higher gaming frequency had a negative impact on offline social capital (Williams, 2006a), while Huvila et al. (2010) found that social capital built within Second Life does not converge with offline social capital. Kowert et al. (2014a) found that gaming frequency seems to be negatively associated with the quality and size of offline social circles. Together, these findings suggest that players who develop in-game relationships spend less time and energy fostering their offline relationships (Kraut et al., 1998; Shen and Williams, 2011; Domahidi et al., 2014; Kowert et al., 2014a) and that in-game social ties might be the broader and weaker “bridging” ties that do not provide the same level of social support provided by in-person relationships (Steinkuehler and Williams, 2006; Williams, 2006a,b; Williams et al., 2006; Huvila et al., 2010; Shen and Williams, 2011; Domahidi et al., 2014); however, these studies have not measured the effects that displacing offline relationships with online ones or investing in bridging ties through gaming have on an individual's loneliness or wellbeing.

Other studies have suggested that digital games—played both in-person and online—can foster the social interactions that are vital for our social well-being. Stereotypes about the antisocial, lonely gamer have long been shown to be inaccurate (e.g., Kowert et al., 2014b; Schiano et al., 2014) and researchers are finding that players view games as a social medium on which they want to form and maintain friendships (Steinkuehler and Williams, 2006; Kowert and Oldmeadow, 2015). Importantly, it has recently been demonstrated that these in-game relationships give out-of-game value to players. For example, Trepte et al. (2012) found that both bridging and bonding social capital built in esports clans was positively associated with offline social support (advice, assistance, and listening). Depping et al. (2018) found that interdependent and benevolent gaming communities facilitated both bridging and bonding capital in games, and that both were associated with reductions in loneliness (and bridging with increases in relatedness satisfaction).

Previous research into the value of social gaming has suggested that players devoting time to engaging in game-based social interactions, or forming and maintaining relationships through social gaming, may result in either value and harm. Most research has explored the nature or characteristics of social play, often by using the theoretical framework of social capital, yet very few studies have explicitly measured its benefits or harms to the wellbeing of players in operationalizations of social embeddedness, such as offline social support (e.g., Trepte et al., 2012), satisfaction of relatedness (e.g., Depping et al., 2018), or loneliness (e.g., Depping et al., 2018). When considering how problematic gaming is facilitated or how the wellbeing of players is affected by play, answers often take the form of “*it depends”*: gaming is not good or evil, but its value depends on the person playing, the game played, and the complex interactions that involve the gaming context (Johnson et al., 2013). One of these important factors of the gamer and their context is their passion orientation—is their passion for gaming an authentic relationship in balance and harmony with other life aspects or is it characterized by preoccupation and an inflexible persistence? And does this passion orientation affect the value of social ties established through social play? And finally, how do these social ties established through passionate play affect the wellbeing of players? In the end, are passionate social gamers more or less lonely?

### 1.4. The Present Study

In this paper, we model several contingent factors that differentiate between beneficial and harmful outcomes within players of the same massively multiplayer online role playing game (MMORPG). We surveyed 300 World of Warcraft (WoW) players, recruited online from fan forums, asking about their passion for playing WoW, their WoW-based social capital, their loneliness, and overall wellbeing (in addition to demographic factors and gaming behaviors). Our goal was to model how passion for playing WoW builds in-game social capital, and then translates into out-of-game experiences of loneliness and wellbeing.

#### 1.4.1. Hypothesized Path Model

**Figure 1:**
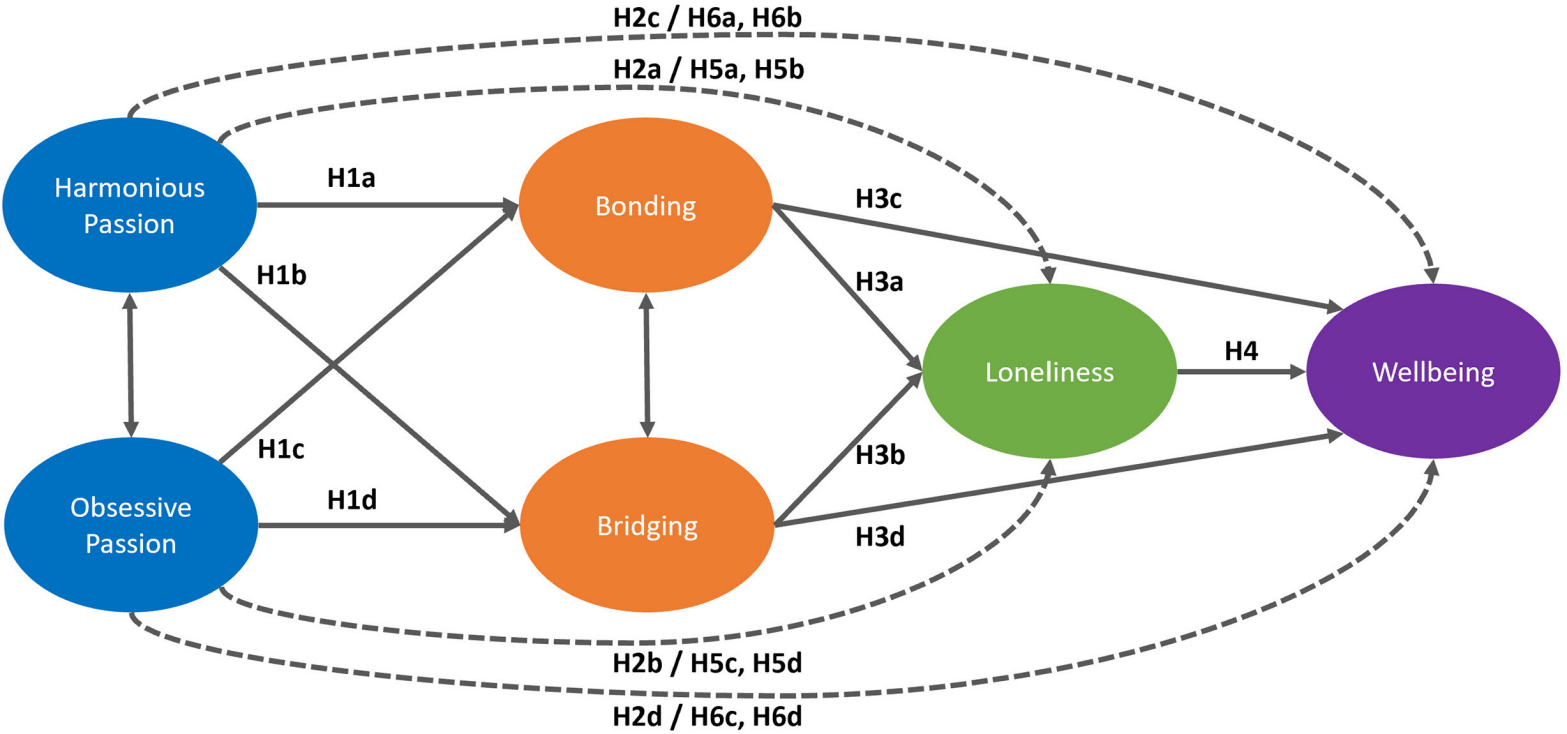
The hypothesized Structural Equation Model. Error terms for the measured items are not shown. Dashed lines indicate that we assumed direct and indirect effects for these paths.

The path model (see Figure 1) was derived from the literature, with support for several hypotheses along with other tested paths.

##### 1.4.1.1. Direct effects

The first hypothesized paths (H1) are concerned with the relationships between passion and social capital. Based on the evidence seen in Perry et al. (2018) that HP leads to both types of social capital, we propose that:

*H1a. Harmonious Passion will be positively associated with in-game Bonding social capital*.
*H1b. Harmonious Passion will be positively associated with in-game Bridging social capital*.

In contrast, we expect that obsessive passion will not be associated with in-game bridging or in-game bonding capital, based on Perry et al. (2018) not having observed these relationships in the context of Destiny. However, because our gaming context is different (WoW), we still test these relationships in the model. Even if a player is obsessively passionate about WoW, they still might reap the benefits of social play. Further, recent work using a quadripartite model of passion (Schellenberg et al., 2019) shows that although HP and OP vary independently, players with mixed passion (high in both HP and OP) can have similar experiences to those with pure passion (either HP or OP). There may be an association; however, we have no guidance on the direction of the association, thus these hypotheses are exploratory, rather than confirmatory.

*H1c. Obsessive Passion will be associated with in-game Bonding social capital*.
*H1d. Obsessive Passion will be associated with in-game Bridging social capital*.

H2 is concerned with the relationships between passion and our outcome variables of Loneliness and Wellbeing. Based on existing research (in both videogame and other contexts) showing increased negative outcomes associated with obsessive passion and increased positive outcomes associated with harmonious passion, we generated H2b and H2c. While the other paths (decreased positive outcomes associated with obsessive passion and decreased negative outcomes associated with harmonious passion) have been found less reliably, based on the work of Przybylski (Przybylski et al., 2009) and Schellenberg (Schellenberg et al., 2019), we generated H2a and H2d.

*H2a. Harmonious Passion will be negatively associated with Loneliness*.
*H2b. Obsessive Passion will be positively associated with Loneliness*.
*H2c. Harmonious Passion will be positively associated with Wellbeing*.
*H2d. Obsessive Passion will be negatively associated with Wellbeing*.

H3 is concerned with the relationships between social capital and our outcomes of loneliness and wellbeing. As previously discussed, there is some dissent on whether in-game social capital translates into reduced loneliness and improved wellbeing; however, many of the studies that suggest this do not explicitly test these relationships empirically. Based on Depping et al. (2018), who found that both in-game bridging and bonding social capital decreased loneliness, we assumed that in-game social capital (both bridging and bonding) would be negatively associated with loneliness and positively associated with wellbeing.

*H3a: In-game Bonding social capital has a negative association with Loneliness*.
*H3b: In-game Bridging social capital has a negative association with Loneliness*.
*H3c: In-game Bonding social capital has a positive association with Wellbeing*.
*H3d: In-game Bridging social capital has a positive association with Wellbeing*.

Based on a wealth of research that suggests there is significant harm to our wellbeing when we experience social isolation and loneliness, we hypothesized that:

*H4: Loneliness has a negative association with Wellbeing*.

##### 1.4.1.2. Mediated effects

We expect that social capital built from harmonious passion is associated with loneliness. Based on Depping et al. (2018), who showed that both bridging and bonding social capital reduced loneliness, and Perry et al. (2018) who showed that HP builds social capital, we assume that the proposed effect of harmonious passion on loneliness is mediated by in-game social capital. We consider mediation effects as indirect effects. As such, we propose:

*H5a. There is a negative indirect effect of Harmonious Passion on Loneliness through in-game Bonding social capital*.
*H5b. There is a negative indirect effect of Harmonious Passion on Loneliness through in-game Bridging social capital*.
*H5d. There is a positive indirect effect of Obsessive Passion on Loneliness through in-game Bonding social capital*.
*H5d. There is a positive indirect effect of Obsessive Passion on Loneliness through in-game Bridging social capital*.

Finally, we assume that the positive effect of harmonious passion on wellbeing is caused by its positive effect on social capital and therefore its effect on loneliness. We assume social capital built from Harmonious Passion will be positively associated with wellbeing and expect that:

*H6a. There is a positive indirect effect of Harmonious Passion on Wellbeing through in-game Bonding social capital and Loneliness*.
*H6b. There is a positive indirect effect of Harmonious Passion on Wellbeing through in-game Bridging social capital and Loneliness*.
*H6c. There is a negative indirect effect of Obsessive Passion on Wellbeing through in-game Bonding social capital and Loneliness*.
*H6d. There is a negative indirect effect of Obsessive Passion on Wellbeing through in-game Bridging social capital and Loneliness*.

## 2. Materials and Methods

To test our assumed model, we gathered data by surveying WoW players, a game that has been used in the earlier research on social capital (Williams et al., 2006) and might therefore be well suited for investigating the contingent factors that lead to positive or negative outcomes.

World of Warcraft (WoW) is a popular Massively Multiplayer Online Role-Playing Game (MMORPG), which had an estimated 5 million players in 2019 (Statista Research Department, 2016), but 12 million active players at its peak in 2010 (Peckham, 2013). WoW is a persistent multiplayer game world in which players can create their own in-game representations, known as a “character” or “avatar,” through which they interact with the digital world as well as with other players. Players can customize their character's appearance, personality, and talents, and can “level” their character (maximum level = 120) by participating in various challenges, which unlock new abilities and equipment. WoW features built-in systems to associate with other players, including through factions and guilds. Additionally, WoW provides activities that can be broadly classified as Player vs. Environment (PvE; players fight together against virtual enemies) or Player vs. Player (PvP; players fight each other). These activities can be played in a group, or solo, and players can selectively engage in or ignore these different aspects of the game. WoW players have multiple ways to socialize. Players have access to different chat channels, such as guild chats, party chats, trade chats, zone chats, nearby chats that only the players in a specific area can see, and direct chats in which one can privately whisper with a certain player. Additionally, non-verbal communication techniques allow players to communicate strategies by drawing instructions on the in-game map, alerting others by pinging, or through player emotes. As described in the introduction, the rich social interactions available within the game have made WoW the subject of broad study.

### 2.1. Survey Design and Measures

We implemented a survey system using an existing framework (Johanson, 2020). Participants connected to a website that was hosted on our servers and guided them through the survey. First, participants provided informed consent and then answered questionnaires about their demographic background and play behaviors and experiences as follows:

**Gaming Expertise:** We assessed general gaming attitudes and expertise with several questions. Participants reported their current and previous average play times by selecting one of five pre-defined categories (from “Never” to “Every Day”), genres they enjoy playing, changes in recent and future schedules that affected regular play behavior, and identity as gamers on a 10-point scale, which has been previously shown to correlate highly with a 60-item questionnaire on gaming identity (Mandryk and Birk, 2017). In terms of WoW-specific measures, participants reported their expertise on a 50-point scale from 1 (= “Novice”) to 50 (= “Expert”) and provided links to armories on worldofwarcraft.com for their main and alternative characters.

**Passion:** We used Vallerand and colleagues' Passion Scale (Vallerand et al., 2003) to measure passion. We adapted the questionnaire to refer to playing WoW, e.g., “Playing World of Warcraft is in harmony with the other activities in my life.” In the resulting scale, participants reported their harmonious (6 items, α= 0.800) and obsessive passion (6 items, α= 0.826) for playing WoW on 7-point scales from 1 (= “Not true at all”) to 7 (= “Very true”).

**Social Capital:** We adjusted the Internet Social Capital Scales (ISCS) (Williams, 2006b) to measure bridging (10 items, α= 0.934) and bonding (10 items, α= 0.897) social capital. All items were measured on 7-point scales from 1 (= “Strongly disagree”) to 7 (= “Strongly agree”). Instructions and items were adapted to refer to WoW, e.g., “Interacting with people from World of Warcraft makes me feel like part of a larger community.” As such, participants reported bridging and bonding social capital for in-game relationships within WoW.

**Loneliness:** We used the revised UCLA Loneliness Scale (Russell et al., 1980) to measure the participants' experience of loneliness with 19 items, answered on a 4-point scale. Participants answered how often they felt the way described in each statement, e.g., “There are people I feel close to.” (1 = “Never,” 2 = “Rarely,” 3 = “Sometimes,” 4 = “Often”). The scale had excellent internal consistency (α= 0.930).

**Wellbeing:** We employed the World Health Organization Five Wellbeing Index (WHO-5) (World Health Organization, 1998; Topp et al., 2015) to measure the participants' wellbeing. It uses five items, e.g., “I have felt cheerful and in good spirits,” rated on 6-point scales from 1 (= “All of the time”) to 6 (= “At no time”). The scale was recoded for easier interpretation in a way that higher scores correspond to higher wellbeing. The internal consistency was good (α= 0.853).

**Gaming-Related Explicit Motives:** We also gathered a number of items related to their motives for gaming; however, this scale was under development, has yet to be validated, and we do not report further on this data in this manuscript.

### 2.2. Participants

We recruited active WoW gamers by advertising on several WoW-related forums, including: www.warcraftpets.com, www.wow-petopia.com, www.icy-veins.com, www.wowhead.com, and www.mmo-champion.com. Following best practices (Mandryk, 2016), the third author posted all of the recruitment materials as she is an active WoW member, has a level-120 ranked character, is visible in posting on forums, and provides credibility and trust to potential participants (in contrast to the other authors who are not active in the online WoW community). She also shared the survey link via twitter and Discord. To compensate participants for their time, we offered five random draw prizes of WoW game time cards (each equivalent to 6 months of play).

We received 319 responses from participants. After filtering out negligent responses (see section 2.3), we retained data from 300 participants (261 men, 38 women, 1 prefer not to answer) aged 18–66 (*M* = 30.7, *SD* = 7.7). In terms of frequency of play, the majority (70%) played WoW daily, whereas 28% played a few times per week; 1.7% played a few times per month and 0.3% played a few times per year. Our participants had high expertise with the game (*M* = 42.1, *SD* = 7.2 on a 50-point scale), and rated themselves as an average of 8.2 (*SD* = 2.0) on a 10-point scale of gamer identity. In sum, our sample consisted of expert and frequent WoW players, who identified as gamers and were predominantly men.

### 2.3. Data Analyses

We first re-coded all reverse-coded items and calculated the means and standard deviations (*SD*) for each scale's subconstructs for each of the 319 respondents. In line with best practices for online data collection (Meade and Craig, 2012; Buchanan and Scofield, 2018), we filtered out responses that did meet threshold quality criteria. First, we removed 3 participants who completed the survey too quickly, defined as less than 1.5 s per item on the UCLA, ISCS, Passion, or GREM surveys, indicating a lack of attention to the items. Second, we removed 3 people who had zero variance on any of the same four surveys, indicating that they simply clicked the same response (e.g., “strongly agree”) throughout. Third, we removed 7 people using a variance filter (Meade and Craig, 2012; Buchanan and Scofield, 2018), defined as people whose standard deviations of responses on a subscale were more than two standard deviations above mean variance on 2 or more subscales (from HP, OP, Bonding, Bridging, Loneliness, and Wellbeing). This pattern of responses indicates that participants were clicking randomly, yielding large variance for a subscale on which responses should be relatively consistent. Finally, we removed 6 people who indicated their age as under 18 or equal to 120 (*n* = 2), which is the highest value accepted by our system. This filtering process removed 19 participants (5.96%) and left 300 valid responses.

### 2.4. Structural Equation Modeling (SEM)

SEM was completed in JASP v 0.12.2, using Lavaan syntax (Rosseel, 2012; JASP Team, 2020) and the MPlus Emulation. We used ordinal items and accordingly used robust DWLS estimation (The lavaan Project, 2016; Gana and Broc, 2019). We estimated one structural equation model, in which Bridging and Bonding Social Capital were used to directly predict Loneliness and Wellbeing, Loneliness predicted Wellbeing, and Bridging and Bonding were directly predicted by HP and OP, which also were used to directly predict the outcome variables. OP was allowed to covary with HP and Bridging with Bonding (see Figure 1). All variables were estimated using manifest item scores (six for each of HP and OP, ten for each of bridging and bonding, nineteen for loneliness, and five for wellbeing).

## 3. Results

**Figure 2:**
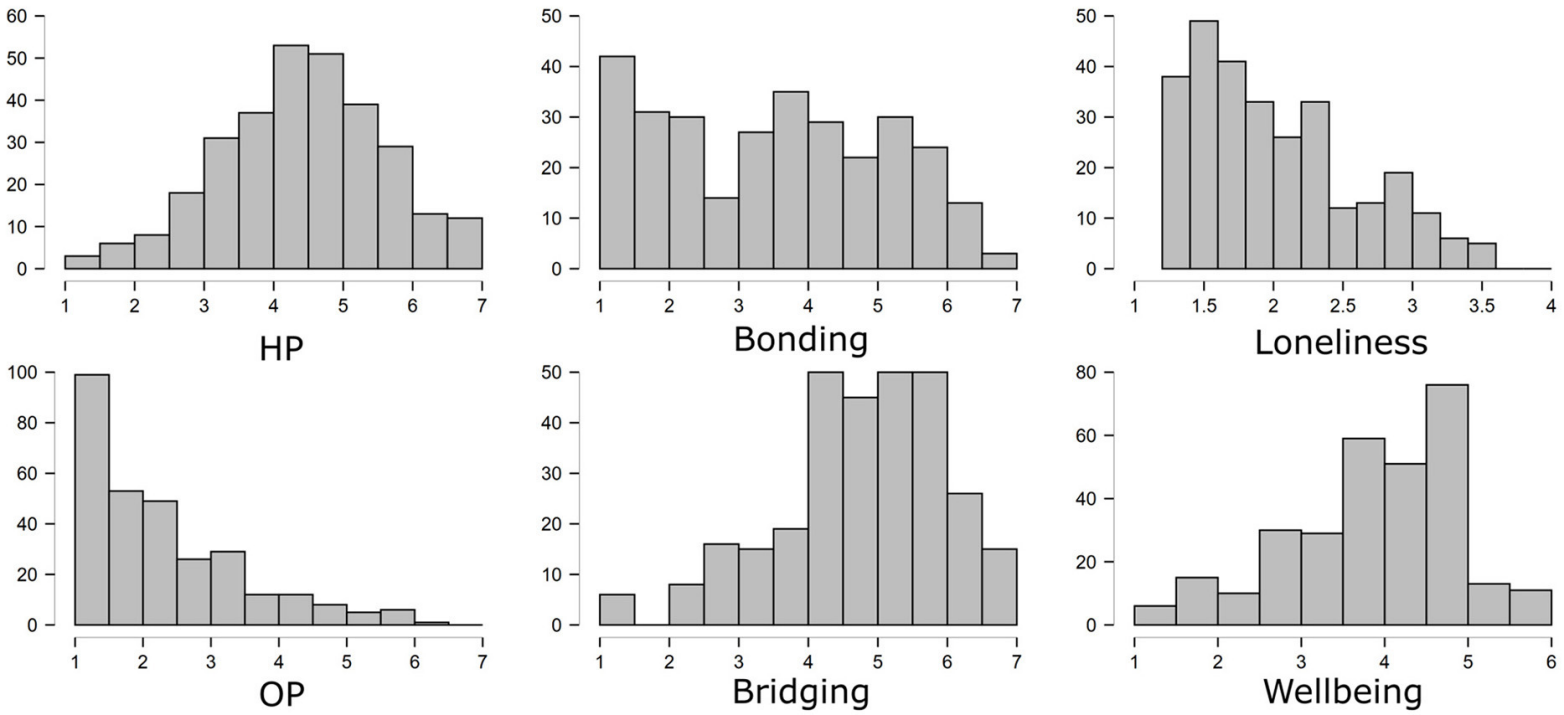
Distributions for harmonious (HP) and obsessive passion (OP), in-game bonding (Bonding) and bridging (Bridging) social capital, loneliness, and wellbeing.

In Table 1, we present the means, standard deviations, and correlations for the variables included in the model. In addition, Figure 2 shows histograms for all constructs to illustrate distribution of responses.

**Table 1:** Means, standard deviations, and correlations for harmonious (HP) and obsessive passion (OP), in-game bonding (Bonding) and bridging (Bridging) social capital, loneliness, and wellbeing.

			***r***					
	***M***	***SD***	**HP**	**OP**	**Bonding**	**Bridging**	**Loneliness**	**Wellbeing**
HP	4.505	1.175	–					
OP	2.342	1.198	0.091	–				
Bonding	3.556	1.602	0.383***	0.129*	–			
Bridging	4.828	1.190	0.513***	0.160**	0.571***	–		
Loneliness	1.981	0.603	−0.232***	0.167***	−0.269***	−0.113	–	
Wellbeing	3.930	1.013	0.266***	−0.093	0.100	0.100	−0.576***	–

*p < 0.05, **p < 0.01, ***p < 0.001.

### 3.1. Structural Model Fit

We examined the fit of our hypothesized model. Following recommendations (Bollen and Long, 1993; Hu and Bentler, 1999; Kenny, 2020), we used a variety of model fit indices with the chi-square statistics, incremental fit index (IFI), comparative fit index (CFI), Root Mean Square Error of Approximation (RMSEA), and Standardized Root Mean Square Residual (SRMR). Overall, the indices suggested satisfactory fit [IFI = 0.975, CFI = 0.975, $\chi_{\left(1469\right)}^{2}$ = 3254.570, *p* < 0.001, χ^2^/df = 2.22, RMSEA = 0.064, SRMR = 0.077].

### 3.2. Path Analysis

We explored the hypothesized relationships between constructs with direct and indirect effects, and report unstandardized path coefficients, denoted by β.

#### 3.2.1. Direct Effects

**Figure 3:**
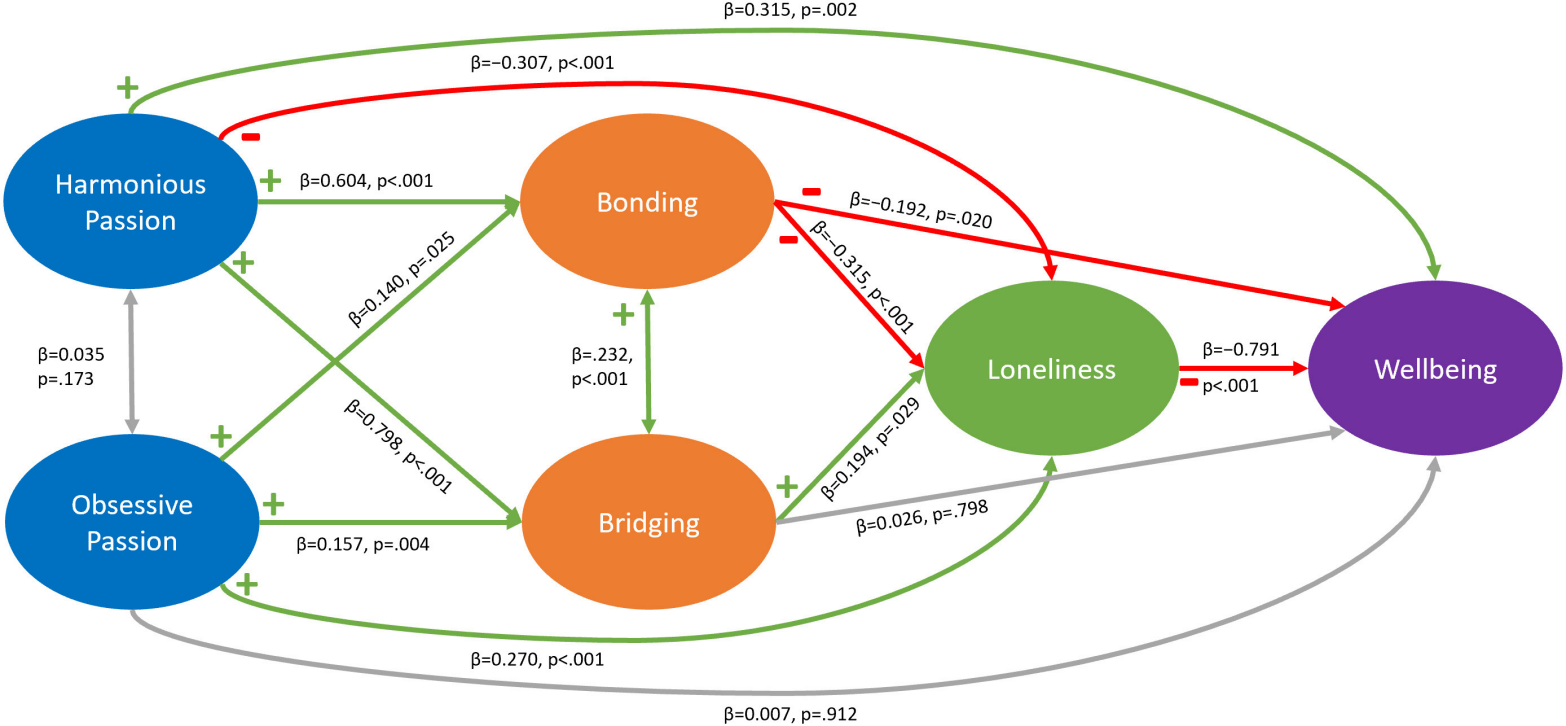
Direct effects for hypothesized paths. β denotes unstandardized coefficients.

Figure 3 shows direct effects in our model.

##### 3.2.1.1. Effects of passion on in-game social capital

In our model, harmonious and obsessive passion were only slightly and non-significantly associated (β= 0.035, *p* = 0.173). As hypothesized (H1a & H1b), the results show that harmonious passion was positively associated with in-game Bonding social capital (β= 0.604, *p* < 0.001) and in-game Bridging social capital (β= 0.798, *p* < 0.001). Similarly, obsessive passion had a significant positive effect on in-game Bonding social capital (H1c, β= 0.140, *p* = 0.025) and in-game Bridging social capital (H1d, β= 0.157, *p* = 0.004), albeit with smaller effects.

These results confirm H1a, H1b, H1c, and H1d.

##### 3.2.1.2. Effects of passion on loneliness and wellbeing

Our model confirmed the hypothesized effects of passion on loneliness and wellbeing only in part. As expected, harmonious passion was negatively associated with loneliness (β= −0.307, *p* < 0.001) and positively associated with wellbeing (β= 0.315, *p* = 0.002). In line with expectations, obsessive passion has a positive association with loneliness (β= 0.270, *p* < 0.001), while its effect on wellbeing was not significant (β= 0.007, *p* = 0.912).

Thus, H2a, H2b, and H2c were confirmed, but we did not find support for H2d.

##### 3.2.1.3. Effects of in-game social capital on loneliness and wellbeing

We tested direct effects of social capital on our outcome variables. First, our model confirmed a positive relationship between Bonding and Bridging social capital (β= 0.232, *p* < 0.001). In line with earlier work (Depping et al., 2018), our results show a negative association between Bonding social capital and loneliness (H3a, β= −0.315, *p* < 0.001). On the other hand, there was a positive relationship between Bridging social capital and loneliness (H3b, β= 0.194, *p* = 0.029), which lies in contrast to earlier work, in which bridging social capital decreased loneliness (Depping et al., 2018), and is the opposite of what our hypotheses predicted. The model showed significance of the direct effect of in-game Bonding social capital on wellbeing (H3c, β= −0.192, *p* = 0.020), while the effect of in-game Bridging social capital on wellbeing was not significant (H3d, β= 0.026, *p* = 0.798).

As such, we confirm H3a and H3c, but find no support for H3d. For H3b, we found the opposite relationship to be significant, thus we cannot confirm H3b.

##### 3.2.1.4. Effects of loneliness on wellbeing

Our model confirmed H4 about the relationship between loneliness and wellbeing; we show a strong negative relationship between loneliness and wellbeing (β= −0.791, *p* < 0.001).

We confirm H4.

#### 3.2.2. Mediated Effects

##### 3.2.2.1. Mediation of effects from passion to loneliness through social capital

As passion affected social capital, which was associated with loneliness, we were interested in whether the effect of passion on loneliness is mediated by social capital. We tested these effects as hypothesized in H5a & H5b, but also tested the same effects for obsessive passion (H5c & H5d). Our model showed a significant negative indirect effect of harmonious passion on loneliness through in-game Bonding social capital (H5a, β= −0.190, *p* < 0.001), while the effect through in-game Bridging social capital was positive and significant (H5b, β= 0.155, *p* = 0.033). This suggests that there is a partial mediation of the effect of harmonious passion on loneliness through social capital while direction seemed to depend on the type of social capital: bonding decreased loneliness while bridging seemed to increase it. The effects had the same direction but were smaller for the marginally significant indirect effect of obsessive passion through Bonding on Loneliness (H5c, β= −0.044, *p* = 0.046) and the not significant effect through Bridging (H5d, β= 0.030, *p* = 0.094). As such, our results suggest that social capital might mediate the effects of passion on loneliness, mostly for harmonious passion and through Bonding.

**Figure 4:**
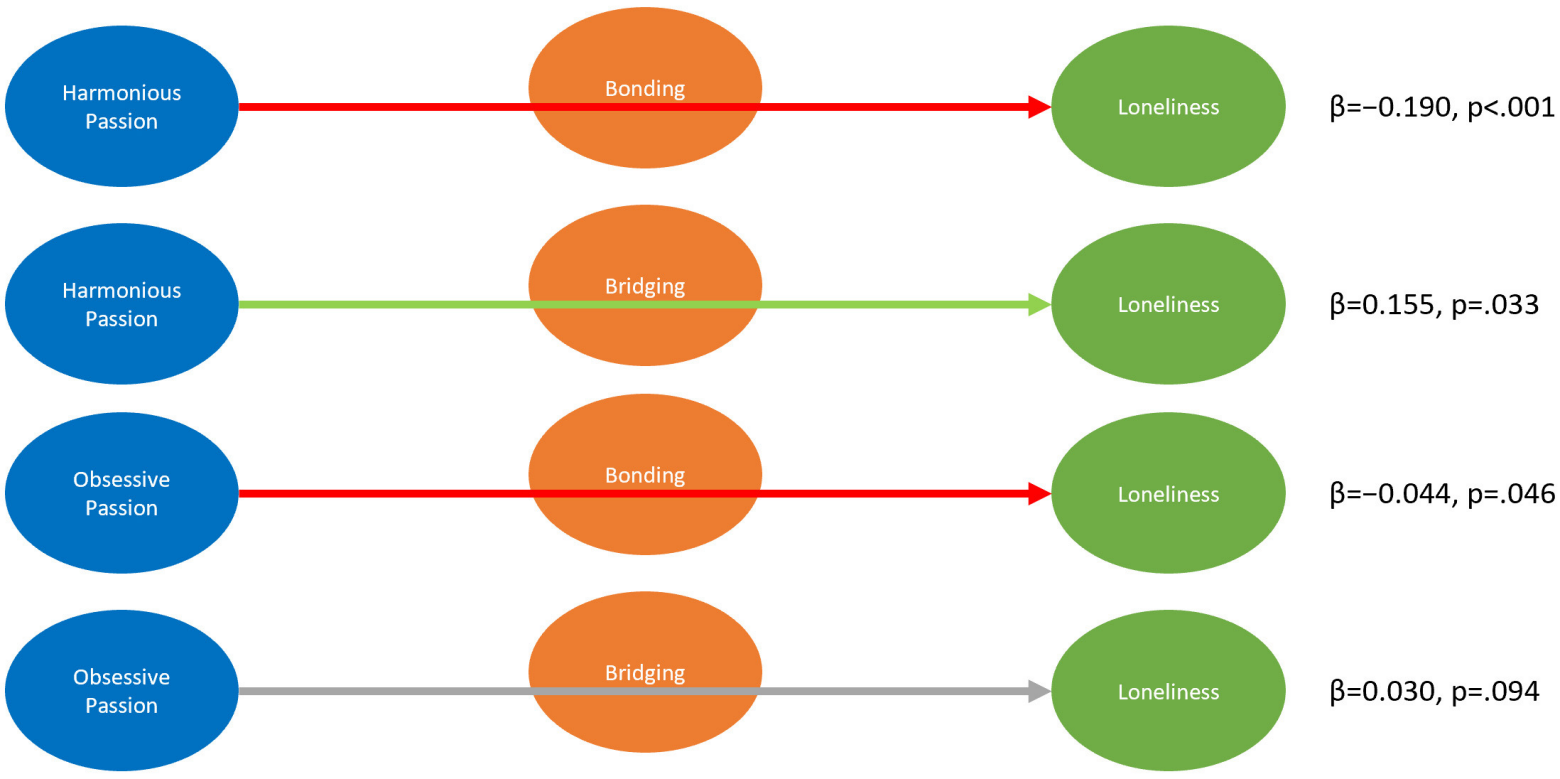
Indirect Effects from Passion on Loneliness mediated by Social Capital. β denotes unstandardized coefficients.

Thus, we confirm H5a, H5b, and H5c. We do not find support for H5d. See Figure 4 for an overview.

##### 3.2.2.2. Mediation of effects from passion to wellbeing through social capital and loneliness

Finally, we were interested in the mediation of the effect of passion on wellbeing. We calculated the indirect effects of passion (Harmonious and Obsessive) on wellbeing mediated by in-game social capital (Bonding and Bridging) and loneliness. The model showed that the indirect effect of harmonious passion through Bonding and Loneliness (β= 0.150, *p* < 0.001) was positive and significant (H6a). On the other hand, the indirect effect of harmonious passion on wellbeing through in-game Bridging social capital and loneliness was negative, albeit marginal in significance (H6b, β= −0.122, *p* = 0.036). For obsessive passion, significance was not reached for the indirect effects through Bonding and Loneliness (H6c, β= 0.035, *p* = 0.053) and through Bridging and Loneliness (H6d, β= −0.024, *p* = 0.096). This suggests that the positive effect of harmonious passion on wellbeing is partially mediated by social capital, mostly through Bonding, and a resulting decrease in Loneliness. On the other hand, the role of Bridging social capital might be more complicated, potentially harming wellbeing even for harmonious passionate play. However, this and the effects for obsessive passion remain inconclusive due to marginal significance and therefore suggest further research.

**Figure 5:**
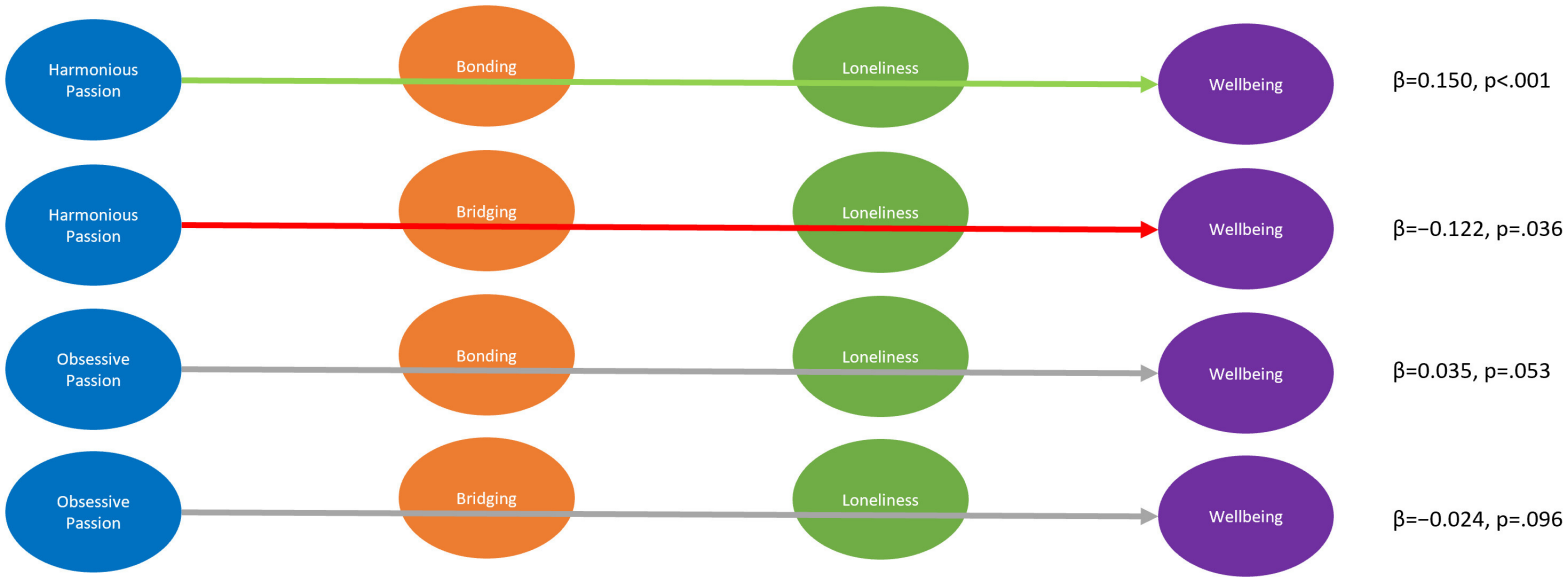
Indirect Effects from Passion on Wellbeing mediated by Social Capital and Loneliness. β denotes unstandardized coefficients.

Thus we confirm H6a, but cannot confirm H6c or H6d. For H6b, we found a significant effect in the opposite direction, thus cannot confirm H6b. See Figure 5 for an overview.

## 4. Discussion

We summarize the results, contextualize them within literature, and discuss implications and limitations.

### 4.1. Summary of Results

Our results can be summarized as follows:

- Passion for WoW is positively associated with both types of in-game Social Capital, with Harmonious being the stronger predictor than Obsessive Passion
- Harmonious Passion for WoW decreases Loneliness and increases Wellbeing
- Obsessive Passion is associated with higher Loneliness, but not directly associated with Wellbeing
- Loneliness is negatively predicted by Bonding Social Capital but positively by Bridging Social Capital
- Bonding Social Capital has a negative direct effect on Wellbeing, while Bridging Social Capital does not directly predict Wellbeing
- Wellbeing is negatively predicted by Loneliness
- Indirect effects suggest that Harmonious Passion increases Wellbeing by building Social Capital (mostly through Bonding), and reducing Loneliness.

### 4.2. Explanation, Comparison, and Implications

#### 4.2.1. On Passion for WoW

Our results provide additional evidence of the importance of passion in understanding the impacts of videogame play on the wellbeing of players, and highlight the potential of passion as a way to both maximize benefit and minimize harm for players. As expected, our results confirmed the pattern seen in prior work in which OP is associated with increased negative outcomes (i.e., loneliness, H2b) and HP is associated with increased positive outcomes (i.e., wellbeing, H2c). Interestingly, in terms of the less consistently confirmed complementary paths, we found support for one of our hypotheses but not the other. Specifically, we found that HP was associated with a reduction in loneliness (H2a) consistent with Schellenberg (Schellenberg et al., 2019) and colleagues (who found a reduction in negative physical symptoms) and Przybylski and colleagues (Przybylski et al., 2009) who found a reduction in post-play tension. However, we found no association between OP and wellbeing, which is consistent with previous work finding OP was not related to self-development nor social interaction (Tóth-Király et al., 2019). Overall, while there is increasingly strong evidence that HP increases positive and OP increases negative outcomes, it is not yet clear under which circumstances and for which outcomes, HP reduces negative and OP reduces positive outcomes. Results are inconclusive and depend on context and what outcome measures are chosen; further research is needed.

Consistent with the research of Perry et al. (2018), who explored passion and social capital in the context of Destiny, we found further support for HP being associated with increased social capital (both bridging and bonding; H1a, H1b). However, in contrast to their findings, we also found OP to be associated with social capital (H1c, H1d), albeit as a much weaker relationship than that found for HP, as evidenced by the relative beta values. While it seems clear from both of these studies that HP is preferable in terms of players benefiting from social connections (both in terms of deep bonds and broader connections), OP may in the context of some games also be associated with beneficial social relationships. However, it is worth highlighting that in explicitly testing the mediated effects in our study, the indirect paths of HP through social capital to the outcome measures were consistently significant, whereas the indirect effects from OP were not.

We also found that, surprisingly, bonding social capital has a direct, negative relationship with wellbeing. This could either be an overestimated null effect given the p-value and estimations for coefficient, whose 95% CI [−0.350, −0.030] is close to including 0. Alternatively, it might be possible that players who have strong in-game social ties might do so at the expense of other activities that also increase wellbeing. Following the indirect effects (H6a & H6b), this negative and direct effect might be counteracted by the strong positive effect of HP on wellbeing through its decrease in loneliness and resulting effect on wellbeing. Further, the strong and positive direct effect of HP on wellbeing and negative direct effect on loneliness reinforce that a harmonious passion for games is associated with increased positive outcomes and decreased negative ones. Together, our results suggest that players who have high harmonious passion for WoW could have resulting benefits on wellbeing through strong bonding social ties within the game and a resulting decrease in loneliness, while we require further research to disentangle the factors under which bonding social capital affects wellbeing.

#### 4.2.2. Is Bridging Capital Harmful in WoW?

Although we found that HP builds both bridging and bonding social capital, bonding is associated with a reduction in loneliness, whereas bridging is associated with increasing it. Further, the indirect effects from HP to loneliness and wellbeing through bonding and bridging reinforce the beneficial effects of bonding capital in that the paths from HP through bonding improve wellbeing and reduce loneliness, whereas the paths through bridging (although weaker and marginally significant) reduce wellbeing and increase loneliness. Why might bridging harm social wellbeing while bonding helps? Players communicate during play using a variety of mechanisms, but also use third-party tools (e.g., Discord) to communicate both synchronously and asynchronously out of the game, sometimes about the game itself and sometimes about non-game-related topics. It is possible that in-game bonding capital is more likely to lead to out-of-game interaction than in-game bridging capital, which might, in turn, provide more social connectedness; however, further work needs to be conducted. Additionally, bridging social capital may not benefit players in terms of affecting their loneliness and wellbeing, but may have other types of social benefits to players, (e.g., widened perspective, global awareness), which we did not measure in our study.

Our results on how in-game bridging capital is associated with social connectedness contrast those of Depping et al. (2018), who found that both in-game bridging and bonding capital built in a broad gaming community decreased loneliness, and those of Trepte et al. (2012), who found that both bridging and bonding social capital built in esports clans was positively associated with offline social support (i.e., advice, assistance, and listening). A potential explanation is that our data was gathered in the context of a specific game (World of Warcraft) and these prior data were gathered in the context of a gaming community and esports clan (respectively) and not tied to a specific game or game genre. It is possible that *bridging* social capital built in WoW is less effective at facilitating social connectedness offline than bridging capital built through other types of games. Further, due to our cross-sectional study design, we cannot infer causality, and it may just be that players who are lonely engage in WoW game features that facilitate building bridging capital. There has been some suggestion that gamers who are high in social anxiety, i.e., a persistent fear of situations in which individuals are exposed to unfamiliar people or to possible scrutiny and negative evaluation by others (Spence and Rapee, 2016), are preferentially attracted to MMORPG games compared to other genres, such as first-person shooter games (Park Jeong Ha, 2016). Investigating further, Dechant et al. (2020) found that MMORPG players who expressed in-game social anxiety tended to avoid high-challenge and highly-social activities in game, but participated in activities related to character advancement, collection, narrative and low-challenge similarly to non-anxious players (Dechant et al., 2020). When considered in light of our results, MMOPRG players who experience social anxiety within games avoid activities that are more likely to build bonding social capital (socializing and working together to overcome a challenging activity), instead focusing on activities that are likely to build bridging social capital (collecting items and engaging in low-challenge activities). In the context of our study, bridging social capital does little to combat loneliness, and may actually exacerbate experienced isolation, putting players with social anxiety (who seem to be preferentially attracted to MMORPGs) at greater risk of harm. The relationships between the player, the game, and the context of play (Johnson et al., 2013) are complex and differentially affect how gaming affects wellbeing; further research is needed to disentangle these complex interdependencies.

Although the role of bridging capital in facilitating or reducing harm remains uncertain, in all cases of prior work and the present study, bonding social capital built in games was constantly and positively beneficial in offline outcomes, suggesting that in-game interactions do have out-of-game benefits to the social wellbeing of players.

#### 4.2.3. Obsessive Passion as a Compensatory Response

Our mediation findings highlight that it is the social connections being made through videogaming that influence a person's loneliness. For players who are obsessively passionate about World of Warcraft, social connections are still formed and maintained, resulting in both bridging and bonding capital. And the bonding capital built from obsessive passion for WoW even marginally reduces loneliness, although it does not strongly translate into wellbeing. However, this indirect reduction in loneliness is not nearly as strong as the direct and positive effect that higher obsessive passion has on loneliness. Our findings suggest that WoW play that is not in harmony with the rest of life can lead to problematic outcomes in terms of social isolation and wellbeing, and that gamers should be aware of their obsessive passion if they wish to avoid the harmful isolating effects of videogaming. Which raises the question, how does obsessive passion for gaming develop and how can it be reduced?

Previous work on the dualistic model of passion (Lalande et al., 2017) has suggested that obsessive passion for an activity is more likely to develop when that activity is the primary means through which a person's psychological needs for competence, autonomy and relatedness (Deci and Ryan, 2000) are satisfied. In other words, a person may begin to compensate for the lack of need satisfaction in other important domains by focusing on the need satisfaction offered through videogame play. In such situations, the person may become over-reliant on videogames and potentially lose control over their playing habits, thus developing obsessive rather than harmonious passion for gaming. Although not tested explicitly, our results can be interpreted in a way that is consistent with Lalande et al. (2017) notion of obsessive passion as a compensatory result. This compensatory context is a potential explanation for our pattern of results, as while we observe that both types of passion lead to social capital, in the case of obsessive passion, the ultimate outcome was increased loneliness. It may be that in our sample, the participants showing greater obsessive passion were doing so in the context of a lack of need satisfaction (including the satisfaction of relatedness) from other sources, and a resulting over-reliance on videogames for building social connections paradoxically limits their effectiveness for this very purpose.

This compensatory response theory is also supported by recent evidence in the context of online gaming. Kowert et al. (2015) used longitudinal data to test whether negative psychosocial outcomes are a cause or a consequence of online videogame play, and found greater support for the notion that players engage with online games to compensate for pre-existing social difficulties than the notion that engaging in online play results is psychosocial harms. Snodgrass et al. (2018) tested the “rich get richer” and “poor-get-poorer” notion of online gaming, which suggests that psychosocially vulnerable individuals erode their wellbeing in online contexts (Kardefelt-Winther, 2014b), whereas healthy individuals prosper in online contexts (Snodgrass et al., 2014). Looking specifically at the wellbeing of MMORPG players, Snodgrass et al. (2018) find that players can compensate for their loneliness through more intensive online videogame involvement, but, as with our results, only when their play allows them to build meaningful social relationships. Snodgrass et al. (2018) go further to suggest that the outcomes for lonely players might depend on how they play—during regular intensive online play, they have the opportunity to bond with others; however, more casual online play may not allow them to signal their mastery, making them less likely to connect socially and thus reinforcing their loneliness. Further, Di Blasi et al. (2020) demonstrate that problematic WoW playing can be a compensatory response for unfulfilled needs, devalued inner selves, or coping with painful mental states for players with vulnerable narcissism. Although Kowert et al. (2015), Snodgrass et al. (2018), and Di Blasi et al. (2020) do not consider passion for gaming, their general findings are consistent with those of Lalande et al. (2017) and Przybylski et al. (2009) in that problematic game use is not simply a factor of game dosage or gaming interest, but is dependent on the gamer's wellbeing outside of the gaming context. Players are more likely to develop obsessive passion when they rely too heavily on a single context (such as videogames) to satisfy their needs (Lalande et al., 2017)—our findings support that where problematic play is occurring (in terms of harmful effects on social connectedness), we should seek to increase harmonious passion and reduce obsessive passion, which may be possible by introducing players to alternative sources of need satisfaction.

We did not make assumptions regarding psychological needs satisfaction of our players, either through videogames or other aspects of life, and did not gather these data; in future work, we will explicitly investigate this potential compensatory response as an explanation for how the two passion orientations both build social capital in games, but that only the social capital built though harmonious passion benefits players' by combating loneliness.

#### 4.2.4. The Social Benefits of Harmonious Gaming

Not all gaming is created equal; the same game, gamer, or gaming context can lead to play that is a restorative and stress-reducing activity and which fosters social connections, or can lead to play as a problematic and isolating endeavor. By unpacking the role of passion in the building of in-game social capital, and it's resulting effects of loneliness and ultimately wellbeing, our findings have implications for gamers and gaming as a pastime.

These results are consistent with other research (e.g., Williams et al., 2006; Yee, 2006; Cole and Griffiths, 2007; Depping et al., 2018), which suggests that the relationships formed through videogames can be just as important and impactful as those formed in non-game settings. For players whose passion for WoW is in balance with other activities in their lives, gaming provides in-game social opportunities that have out-of-game benefits to social connectedness, which is one of the biggest threats to wellbeing in developed nations. As has been expressed by others previously (e.g., Trepte et al., 2012; Depping et al., 2018; Kowert and Kaye, 2018; Perry et al., 2018), it is past time to throw away the stereotype of gaming as a lonely and socially-isolating activity, and embrace the tangible benefits that can be provided by social gaming as a collective leisure endeavor.

The Entertainment Software Association's (ESA) report on the essential facts about the computer and video game industry in 2019 (Entertainment Software Association, 2019) notes that compared to average Americans, gamers in the USA are as likely to get the same amount of sleep at night, vacation internationally, exercise, go camping or hiking, be civically engaged, have a creative hobby, play an instrument, be vegetarian, and meditate regularly. Gaming as a hobby is neither good nor bad, and the answer of whether gaming leads to harm depends. In our work, we show that it depends on harmonious passion, which is needed to build social capital, combat loneliness, and improve wellbeing. Harmonious passion is about an activity being in balance with the rest of a person's life. Considering the aforementioned ESA report, it is clear that gaming is often part of a balanced life. And when it is (in the context of WoW), massively passionate players benefit greatly through the relationships they form and maintain, and the social connections that those relationships provide.

#### 4.2.5. Limitations and Future Work

Although we provide new insights into the relationships between passion orientation, social capital, and loneliness, our study has several limitations that should be addressed by future research.

This study examined our research questions only in a single game. As such, our findings might be limited to the specifics of WoW and its natural population of gamers. It might be that specific game features affect how players engage with the game and with other players, for example, by facilitating bridging social capital more so than bonding social capital for specific types of players. The relationships between constructs that we model might be specific to WoW, might not generalize well to other games, and therefore should be replicated in a different game context. Other multiplayer game genres (e.g., Multiplayer Online Battle Arenas, Battle Royale) have very large player bases, and very different competition structures and communication channels, suggesting that future work needs to examine these game genres specifically.

Further, our sample was quite biased in terms of gender and play expertise, likely resulting from the sampling approach of posting the survey on WoW forums. The majority of our players identified as men who played WoW daily and reported high expertise. As such, we cannot estimate how well our results replicate for players who do not identify as men and our results might be specific to seasoned WoW players who have an existing passion for, and social ties within, the game. Related, our sampling method did not yield respondents who were low in both HP and OP, limiting us from exploring the quadripartite model of passion described in section 1.2; we have data from participants with pure OP, pure HP, and mixed passion, but not from those with no passion. A new player with lower passion and social capital within the game likely would have a different experience, which could lead to different results. While likely reflective of the active player base for WoW, the results might be specific to our sample and not reflect how these constructs interact for other player groups in WoW, other MMO games, or even other game genres. This echoes the previously stated need for replication within another context, but also with another population within WoW.

In this paper, we specified a model for the relationship between the passion, social capital, loneliness, and wellbeing constructs. While our modeling approach was guided by hypotheses derived from earlier research, there was no existing model that combined all these constructs. As such, even if the majority of individual paths are grounded in literature, the model might be considered exploratory in the context of some paths, e.g., for the effect on obsessive passion on social capital, and for the analysis of the interactions between modeled constructs, such as the mediation effects of social capital and loneliness for the effects of passion on wellbeing. This calls for more confirmatory research examining the relationship between passion, social capital, loneliness, and wellbeing.

We conducted a cross-sectional study, in which we measured all constructs at the same time. While we modeled directed paths based on earlier research that guided our hypotheses, we cannot infer causation. Our findings suggest how passion, social capital, loneliness, and wellbeing might be connected for players of WoW by applying and confirming assumed theoretical relationships that make sense for our data. However, our results cannot confirm the causal path, which suggests the need for further experimental or longitudinal research that can demonstrate causality.

In this paper, we investigated how harmonious and obsessive passion might be used to differentiate between positive and negative outcomes of play. However, passion for games (similar to other activities) does not exist in isolation. As noted in the discussion on obsessive passion as a compensatory response, it might be possible that our results were affected by external factors that lead to different types of passion for play. In the context of problematic gaming, factors such as demography, life circumstances, or even underlying personality disorders (Di Blasi et al., 2020) or mental health concerns, such as anxiety or depression (Mandryk and Birk, 2017), have demonstrated relevance. As such, future research that investigates how passion for games is built will help further understand how passion interacts with other individual characteristics, underlying pathologies, and contexts to affect the outcome of play.

### 4.3. Conclusions

Gaming as a hobby continues to evolve and the demographics of who engages in videogames shifts and changes as gaming becomes more accessible through new devices for playing (e.g., smartphones), mechanisms to purchase games (e.g., online application stores), and opportunity to engage differently with game content through spectator and fan-based interaction (e.g., watching streamers or esports tournaments). Social play—playing with others in person or online—continues to gain popularity, with players joining their friends, family, colleagues, or strangers in joint play. These social interactions have been suggested to both benefit players by fulfilling our fundamental needs of belonging and relating to others, but have also been suggested to be an impoverished form of social interaction that harms players by displacing richer in-person interactions. Recent work consistently shows that neither of these interpretations tells the entire story, and that whether social play results in benefits or harms depends on a variety of contingent factors related to the player, the game, and the gaming context. In this paper, we aim to understand the role of several of these contingent factors in a popular social game by modeling the relationships between passion orientation (harmonious and obsessive), social ties (bridging and bonding), loneliness, and wellbeing.

Our work shows that harmonious passion for WoW is associated with increased bonding capital, reduced loneliness, and improved wellbeing, whereas obsessive passion for WoW also builds social capital, but that these social ties do not have out-of-game benefits to players, and may even result in poorer outcomes. Further, we differentiate the effects of bridging and bonding ties, which again further contributes to the conversation on how the quality of in-game social interactions matters. We demonstrate that passion orientation is important for characterizing the relationship between gaming and social wellbeing, showing that being massively passionate for a social game can provide tangible benefits to social wellbeing, established through in-game social ties, so long as that passion is in harmony with other aspects of the player's life. Our work supports the nuanced perspective that digital gaming can be both a problematic activity that may require intervention, and an appealing leisure activity that provides enjoyment, recovery, and meaningful social interaction for the millions of players who benefit from its captivation.

## Data Availability Statement

The raw data supporting the conclusions of this article will be made available by the authors, without undue reservation.

## Ethics Statement

The studies involving human participants were reviewed and approved by The Behavioural Research Ethics Board at the University of Saskatchewan. The patients/participants provided their written informed consent to participate in this study.

## Author Contributions

RM led the research, gathered the dataset, formulated the hypotheses, helped analyze the data, and wrote/edited the paper. JF formulated the hypotheses, analyzed the data, and wrote and edited the paper. AA helped gather the dataset, and edited the paper. DJ formulated the hypotheses, helped analyze the data, and wrote/edited the paper. All authors contributed to the article and approved the submitted version.

## Conflict of Interest

The authors declare that the research was conducted in the absence of any commercial or financial relationships that could be construed as a potential conflict of interest.
